# Effects of compassion training on brain responses to suffering others

**DOI:** 10.1093/scan/nsab052

**Published:** 2021-05-05

**Authors:** Yoni K Ashar, Jessica R Andrews-Hanna, Joan Halifax, Sona Dimidjian, Tor D Wager

**Affiliations:** Department of Psychology and Neuroscience, University of Colorado Boulder, Boulder, CO 80309, USA; Weill Cornell Medical College, New York, NY 10075, USA; Department of Psychology, University of Arizona, Tucson, AZ 85721, USA; Upaya Institute and Zen Center, Santa Fe, NM 87501, USA; Department of Psychology and Neuroscience, University of Colorado Boulder, Boulder, CO 80309, USA; Renee Crown Wellness Institute, University of Colorado Boulder, Boulder, CO 80309, USA; Department of Psychology and Brain Sciences, Dartmouth College, Hanover, NH 03755, USA

**Keywords:** compassion training, empathy, mindfulness, placebo, burnout

## Abstract

Compassion meditation (CM) is a promising intervention for enhancing compassion, although its active ingredients and neurobiological mechanisms are not well-understood. To investigate these, we conducted a three-armed placebo-controlled randomized trial (*N *= 57) with longitudinal functional magnetic resonance imaging (fMRI). We compared a 4-week CM program delivered by smartphone application with (i) a placebo condition, presented to participants as the compassion-enhancing hormone oxytocin, and (ii) a condition designed to control for increased familiarity with suffering others, an element of CM which may promote compassion. At pre- and post-intervention, participants listened to compassion-eliciting narratives describing suffering others during fMRI. CM increased brain responses to suffering others in the medial orbitofrontal cortex (mOFC) relative to the familiarity condition, *p *< 0.05 family-wise error rate corrected. Among CM participants, individual differences in increased mOFC responses positively correlated with increased compassion-related feelings and attributions, *r *= 0.50, *p *= 0.04. Relative to placebo, the CM group exhibited a similar increase in mOFC activity at an uncorrected threshold of *P *< 0.001 and 10 contiguous voxels. We conclude that the mOFC, a region closely related to affiliative affect and motivation, is an important brain mechanism of CM. Effects of CM on mOFC function are not explained by familiarity effects and are partly explained by placebo effects.

## Introduction

Compassion is a vital societal and interpersonal process that facilitates caring and cooperative behavior (Goetz *et al.*, 2010) and is widely recognized as a virtue across religious and ethical frameworks. Recently, scientific interest has turned toward the cultivation of compassion. Evidence is accumulating that compassion-focused interventions yield benefits for the self and others in community samples (Galante *et al.*, 2014; Quaglia *et al.*, 2020), mitigate burnout in medical providers (Van Berkhout and Malouff, 2015) and improve symptoms and functioning in several patient populations (Hofmann *et al.*, 2011; Gilbert, 2014; Darnall, 2015; Shonin *et al.*, 2015; Berry *et al.*, 2020).

Many compassion training programs have been based on compassion meditation (CM), a secular meditation practice with Buddhist origins. In the scientific literature, CM has been viewed as a practice that influences appraisals of suffering others and consequent emotional responses (Ashar *et al.*, 2016a; Weng *et al.*, 2017), leading to increased empathic care, vicarious optimism, tenderness and other positive (but not negative) affective responses to suffering others (Klimecki *et al.*, 2012; Galante *et al.*, 2014; Ashar *et al.*, 2016b; Kok *et al*., 2016; Koopmann-Holm *et al.*, 2020; Sirotina and Shchebetenko, 2020). CM-based interventions have also been found to promote a range of helping behaviors such as charitable donations and offering one’s seat to a suffering person on crutches (Condon *et al.*, 2013; Weng *et al.*, 2013, 2015; Lim *et al.*, 2015; Ashar *et al.*, 2016b; Böckler *et al.*, 2018). At the biological level, CM-based interventions have been found to reduce inflammation (Pace *et al.*, 2009, 2012) and enhance parasympathetic activity (Bornemann *et al.*, 2016), suggesting potential health benefits as well.

Multiple distributed brain systems are known to support empathy, compassion and altruism. These span cortical networks and subcortical structures that enable inferences about others’ mental states, simulation of other’s embodied experiences, the valuing of others’ welfare and more (De Waal, 2008; Shamay-Tsoory *et al.*, 2009; Decety *et al.*, 2012; Zaki and Ochsner, 2012). Previous investigations of CM have highlighted a key role for two brain systems in particular. One line of research has consistently found that CM engages a mesolimbic pathway, including the nucleus accumbens (NAc), ventral tegmental area (VTA) and medial orbitofrontal cortex (mOFC). These regions show a heightened response to suffering others following a CM intervention, are engaged by compassionate reappraisal of suffering and exhibit cortical thickening among expert CM practitioners (Klimecki *et al.*, 2014; Singer and Klimecki, 2014; Engen and Singer, 2015b; Engen *et al.*, 2018). This pathway is closely tied to positive affect, value and motivation (Haber and Knutson, 2010; Berridge and Kringelbach, 2015) and more broadly to the use of conceptual processes to assign personal meaning to events (Roy *et al.*, 2012; Barrett, 2017; Ashar *et al.*, 2017b). In the interpersonal context, this system has been linked to empathic care, affiliation, interpersonal closeness and vicarious reward (Koenigs *et al.*, 2007; Mobbs *et al.*, 2009; Hare *et al.*, 2010; Krienen *et al.*, 2010; Inagaki and Eisenberger, 2012; Meyer *et al.*, 2013; Moll *et al.*, 2014; Morelli *et al.*, 2015; Ashar *et al.*, 2017a). This suggests that CM may enhance compassion by increasing the value placed on others’ welfare and/or by increasing affiliative affect, perceived interpersonal closeness and related processes (Goetz *et al.*, 2010; Zickfeld *et al.*, 2019).

In distinction, other studies have reported effects of CM primarily in the dorsomedial prefrontal cortex (dmPFC) and temporal–parietal junction (TPJ), including heightened responses to suffering others and cortical thickening following CM-based interventions (Lutz *et al.*, 2008; Mascaro *et al.*, 2013; Weng *et al.*, 2013; Valk *et al.*, 2017; Favre *et al.*, 2020). These regions—key components of the default mode network—support perspective-taking and inferences about others’ internal states (Saxe and Kanwisher, 2003; Buckner and Carroll, 2007; Tankersley *et al.*, 2007; Shamay-Tsoory *et al.*, 2009; Zaki and Ochsner, 2012), suggesting that CM may enhance compassion by promoting these and related processes.

Based on these two sets of findings, a recent debate has focused on whether CM primarily acts on affective or cognitive processes (Dahl *et al.*, 2015, 2016; Engen and Singer, 2015a)—although these likely interact to support compassion (Kanske *et al.*, 2016). We thus focused our analyses on these two sets of regions, to better understand how CM engages brain pathways more closely related to affiliation and affect *vs* those more closely related to mentalizing and perspective-taking.

A central challenge in the study of CM has been identifying and controlling for ‘non-specific’ factors such as engagement in a regular practice, familiarity with and attention to suffering others and expectations of heightened compassion. While a recent large-scale study found specific effects of CM *vs* other meditation practices on several compassion-related outcomes (Klimecki *et al.*, 2014; Kok and Singer, 2017; Singer and Engert, 2019), other studies have found relatively weak support for this, showing little to no difference between CM and active control on compassion-related outcomes (Condon *et al.*, 2013; Galante *et al.*, 2014; Kreplin *et al.*, 2018; Koopmann-Holm *et al.*, 2020). A potentially important, understudied factor is the meditator’s expectations of increased compassion—and in the laboratory context, perceived researcher expectations of increased compassion (demand characteristics). These processes are known to influence empathy and helping behaviors (Rutgen *et al.*, 2015; Rütgen *et al.*, 2015; Nook *et al.*, 2016), but it is unclear whether commonly used control conditions for CM (e.g. mindfulness meditation interventions) are matched on expectations of increased compassion. Another non-specific element of CM is the increased familiarity with suffering others created by meditating on them during CM practice. This might increase awareness of suffering others or influence preferences toward familiar individuals, leading to increase compassion (Zajonc, 2001; Loewenstein and Small, 2007). Comparison conditions controlling for expectations, demand characteristics and increased familiarity with suffering others are needed to better understand the ‘active ingredients’ of CM.

Here, we conducted a three-armed placebo-controlled randomized controlled trial of CM, with each group receiving a 4-week intervention delivered daily by a mobile iPod application. The CM program aimed to teach participants skills for staying engaged with others’ suffering without becoming emotionally overwhelmed (Halifax, 2011, 2012), since engaging with others’ suffering can tax cognitive and emotional resources (Zaki, 2014; Cameron, 2017; Cameron *et al.*, 2019). A comparison group received a placebo intervention—a nasal spray described to them as the compassion-enhancing hormone oxytocin—although it was actually saline. A third group simply listened to brief narratives of suffering others daily and answered factual questions. To investigate the neurobiological effects of CM, we collected functional magnetic resonance imaging (fMRI) pre- and post-intervention.

Intervention effects on self-reported and behavioral outcomes from this trial have been previously reported (Ashar *et al.*, 2016b). The goal of this article was to test the effects of CM on the brain regions of interest described above, with a secondary goal of testing CM effects on recently developed multivariate patterns of brain function related to empathic emotions (Ashar *et al.*, 2017a), described further below.

## Method

### Participants

Out of 311 participants screened for eligibility, 71 healthy adults completed the baseline assessment between January and September of 2012. To be eligible, participants were required to self-report no history of major psychiatric illness, current mental health conditions or breast-feeding (to maintain the oxytocin placebo deception). Standard fMRI exclusion criteria were applied (e.g. no metal in the body and no claustrophobia). Participants were also required to have no previous experience with CM or Loving-Kindness Meditation and at least moderate interest in meditation, as we sought to investigate the effects of CM among healthy, interested novices (e.g. see also Segal *et al.*, 2010; Williams *et al.*, 2014). We also excluded participants who reported in advance that they would not be willing to donate any participation earnings to charities. An additional research aim of ours, orthogonal to the questions addressed in the present manuscript, was to identify within-person behavior and brain predictors of charitable donations (results described in Ashar *et al.*, 2016b, 2017a). Since this requires within-person variability in donation amounts, we excluded participants likely to have no variance in this measure.

Thirteen participants who completed the baseline assessment were not eligible for randomization for a variety of technical reasons (excessive head motion during the baseline fMRI scan: *n *= 7; lost to follow-up: *n* = 2; no donations made: *n *= 2; neurological anomaly discovered at baseline scan: *n* = 1 and not interested in continuing the study, *n *= 1). Thus, *N* = 58 participants were randomized using a computer-generated randomization list, stratified by sex. One participant refused randomization to the placebo oxytocin condition due to unwillingness to use a nasal spray. Thus, the final sample included *N *= 57 analyzed participants, including *N = *36 females, with *M*_age_ = 29.11 years, SD_age_ = 6.35 years, *M*_Subjective SES_ = 6.25 out of 10 and SD_Subjective SES_ = 1.65. Subjective Socioeconomic Status (SES) was measured with a single-item measure (Adler *et al.*, 2000). Additionally, one participant misunderstood the donation task instructions and was dropped from analyses of the charitable donation outcomes. Experimenters were blind to the participants’ assigned intervention for the pre-intervention assessment but not for the post-intervention assessment. Participant demographics and baseline characteristics are provided for each intervention condition in Table 1. Sample size was dictated by the available research funds.

**Table 1.** Participants’ demographics

	CM	OxyPla	Familiarity
Sample size (*n)*	21	18	18
Sex: *n* female (% female)	14 (67)	11 (61)	11 (61)
Age (years): *M* (*SD*)	28.72 (6.83)	27.43 (4.02)	29.63 (7.45)
Subjective SES	6.10 (1.71)	6.24 (1.56)	6.65 (1.50)
Race: *n* White (% White)	17 (81)	15 (83)	14 (78)

Subjective socioeconomic status (SSES) was measured by the MacArthur Scale of SSESS, a 10-point Likert scale ranging from lowest to highest SES (Adler *et al.*, 2000).

Participants were compensated $100 for each MRI session and an additional $1 for each day of the intervention that they completed. After completion of the study, participants in the placebo condition completed a questionnaire assessing the strength of their belief that they were actually taking oxytocin and were then debriefed regarding the nature of the deception and its purpose. The University of Colorado Institutional Review Board approved all procedures, including informed consent. No serious adverse events resulted from any of the intervention conditions.

### Materials and procedures

#### Brain responses to suffering others.

To assess compassion-related brain function, we designed a task resembling daily-life encounters with suffering others. During fMRI scanning conducted before and after the 4-week intervention, participants listened to 24 randomly ordered biographies describing a range of true stories of suffering people such as orphaned children, adults with cancer and homeless veterans. To increase ecological validity, biographies were created from factual information posted on charity websites and then were recorded by one member of the research team as audio segments 26–33.5 s in duration. An authentic facial photograph of each person, also drawn from the charity website, was displayed alongside the audio biography. The people described in the biographies were balanced on age (child or adult), race (Black or White) and sex. Real stories and photographs were used to increase ecological validity. An example biography is: ‘Jessica’s father abandoned his family and her mother was unable to support them alone, so they had to move into a homeless shelter. The shelter provided Jessica’s mother with professional training and childcare. Eventually, the family moved into subsidized housing. Jessica and her sisters have been tremendously supportive of each other. Jessica has managed to stay in school and will finish the year with her class’. To hear this biography while viewing a sample face photograph, as presented to our subjects, visit https://canlabweb.colorado.edu/files/jessica.mp4 (image copyright CC BY-SA 2.0). The text of all biographies is listed in Supplementary Table S1 of Ashar *et al.*, (2017a), and audio and video recordings of all biographies are available for download at https://github.com/canlab/Paradigms_Public/tree/master/2016_Ashar_Empathy_CompassionMeditation. After each biography, participants provided ratings of empathic care or empathic distress (data not presented here). Given the limited time during functional imaging, we collected ratings of other relevant feelings and attributions immediately after the scan, described below.

After this first task in the scanner, participants then heard abbreviated ‘reminder’ biographies (8–11 s) during fMRI scanning while viewing the face photograph of that person. An example reminder is: ‘Jessica’s father abandoned his family. Her mother and sisters moved into a homeless shelter, which provided job training and childcare. Jessica will finish school this year with her class’. Reminders were provided because pilot studies showed that participants had difficulty distinctly recalling each of the 24 biographies when asked to make donation decisions. Following each reminder, participants were given an option to donate a portion of their own experimental earnings to a charity helping that person, from $0 to $100 in $1 increments, as a measure of compassionate behavior. Between trials, participants were asked to press a button indicating the direction an arrow was pointing (left or right); this served as a non-social baseline comparison task. The duration of this inter-trial baseline task was jittered across trials, from 3 to 9 s. During the task, participants were asked to simply listen to the biographies, and CM participants at the post-intervention assessment were asked not to engage in CM while listening to the biographies, for greater comparability across conditions.

This task was completed over three fMRI runs of listening to biographies and rating feelings, followed by two runs of listening to biography reminders and making donations. The task is publicly available at https://github.com/canlab/Paradigms_Public/tree/master/2016_Ashar_Empathy_CompassionMeditation.

#### Self-reported and behavioral measures of compassion.

Primary self-reported and behavioral outcomes were charitable donations, as described in the fMRI task above, and compassion-related feelings and attributions. These ratings included attributions of blame-worthiness for one’s suffering, attributions of how much a person would be benefitted by efforts to help them, feelings of distress and tenderness and perceived similarity both in socioeconomic status and in values and interests. In prior work (Study 1 in Ashar *et al.*, 2016b), we reported that a linear combination of these feelings and attributions, termed ‘Feeling–Attribution–Similarity’ (or FAS) scores, was strongly predictive of charitable donation. We applied this model to the data collected in this study, to generate FAS scores for each participant at pre- and post-intervention. Participants provided ratings on these feelings and attributions on a visual analog scale ranging from ‘not at all’ to ‘extremely’. These ratings were provided after listening to the biographies a second time after scanning, as there was insufficient time during scanning to collect all ratings on all stimuli.

#### Interventions.

After the baseline session, participants were randomized to one of the three interventions—Familiarity, Placebo Oxytocin (OxyPla) or CM training—with *N*_CM_ = 21, *N*_OxyPla_ = 18 and *N*_familiarity_ = 18. The three interventions were delivered via iPod Touch applications developed by the study team and matched across conditions on structure and style. All participants were asked to complete a daily task for 4 weeks on the iPod Touch provided to them. A member of the study team placed three phone calls to participants during the intervention to address any concerns, ask about side effects in the OxyPla condition and encourage compliance.

Participants in all three conditions listened to a biography of a suffering person every day while viewing a photograph of that person. Out of the 24 total biographies presented during the fMRI sessions, each participant listened to and viewed a set of 12 biographies across the 4-week intervention period. The set of biographies presented to each participant during the intervention was randomly assigned and balanced across groups.

#### Compassion meditation.

The CM program was designed to enhance both compassion for suffering others and equanimity. The emphasis on equanimity aimed to help prevent emotional overwhelm from others’ suffering, thereby promoting sustainable compassionate responding (Halifax, 2012). A theme of the meditation recordings was ‘soft front, strong back’, which served as both a metaphor and an embodied approach to being sensitive to and engaged with others’ suffering while remaining emotionally grounded. Meditations included a focus on grounding in the body and connection with the earth as a foundation, perspective-taking practices (e.g. imagine what this person may be experiencing), visual imagery (e.g. imagine the suffering person as a small child and *tong-len* practice) and repetition of compassion-related phrases directed toward the suffering person (e.g. ‘may you find peace’). The meditations also asked participants to direct these practices toward loved ones, for whom it is often easier to generate compassion, and toward themselves, to enhance self-compassion. Participants were asked to practice meditation for about 20 min daily and were provided with a new guided meditation practice at the start of each of the 4 weeks. At a specified point during each daily meditation, participants would hear one of the biographies described in the fMRI task above and were asked to meditate on that person specifically. A description of each weekly CM practice is provided in Table 2. The meditation scripts and recordings are publicly available at https://github.com/canlab/Paradigms_Public/tree/master/2016_Ashar_Empathy_CompassionMeditation/CM_audio_scripts.

**Table 2.** Description of the compassion meditation program

Week	Elements
Elements present in all weeks	Grounding in mindful awareness of breath and body sensations. The refrain ‘soft front, strong back’ as an embodied metaphor for equanimity and compassion
Week 1	Generate compassion for oneself, a close other and the person described in the story using short phrases (e.g. ‘may you be safe’ and ‘may you be free from suffering’)
Week 2	Imagine yourself as a young, happy child, innocent and blameless. Imagine a suffering other as a young, happy child, innocent and blameless, to generate compassion
Week 3	Take the perspective of a close other who is suffering. Imagine their history of lived experience and the current difficulties they may be experiencing
Week 4	*Tong-len.* Breathe in the suffering of another person, visualized as hot smoky air. Exhale relief and healing back to the suffering person, visualized as cool clean air

#### Placebo oxytocin intervention.

The OxyPla intervention was designed to control for placebo effects related to CM, such as (i) participant expectations of increased compassion naturally created by CM and (ii) demand characteristics created by completing a CM intervention in a research context (e.g. wanting to satisfy perceived researcher objectives by exhibiting increased compassion). Every day, participants were instructed to inhale a nasal spray labeled as oxytocin, press a button in the smartphone application confirming they did so and then listen to the daily biography. Participants were also provided with scientific information sheets describing oxytocin’s ability to enhance compassion. Both the OxyPla and Familiarity conditions required ∼2 min each day. The OxyPla intervention materials, including information sheet and nasal spray bottle labels, are publicly available at https://github.com/canlab/Paradigms_Public/tree/master/2016_Ashar_Empathy_CompassionMeditation/placebo_oxytocin_materials.

#### Familiarity intervention.

Participants in the Familiarity condition simply listened to one biography of a suffering person daily. This condition was designed to control for the increased familiarity with suffering others inherent in the CM practice, as familiarity with suffering people could increase liking and enhance compassion (Zajonc, 2001; Loewenstein and Small, 2007). Alternately, this intervention could be viewed from a lens of repeated exposure, which may dampen emotional reactivity over time.

#### Daily attention-to-task check.

After each daily intervention task, participants in all conditions responded to a multiple-choice question designed to test whether they had adequately paid attention to the biography. Participants were asked to indicate the primary hardship afflicting the individual described in the biography they had heard that day (e.g. ‘What was Robert’s primary hardship? (i) AIDS, (ii) cancer or (iii) homelessness’). Participants also provided ratings of mood each day (data not presented here).

### Analyses

#### fMRI data acquisition and preprocessing.

Images were acquired with a 3.0T Siemens Trio Tim MRI scanner using a 12-channel head coil. Twenty-six 3.0 mm-thick slices (in-plane resolution 3.4 × 3.4 × 3.0, 1 mm gap, ascending sequential acquisition) extended axially from the mid-pons to the top of the brain, providing whole-brain coverage (Repetition Time (TR) = 1.3 s, Echo Time (TE) = 25 ms, flip = 75°, field of view = 220 mm, matrix size = 64 × 64 × 26). High-resolution structural scans were acquired prior to the functional runs with a T1-weighted MP RAGE pulse sequence (TR = 2530 ms, TE = 1.64 ms, flip = 7°, 192 slices, 1 × 1 × 1 mm). Parallel image reconstruction (GRAPPA) with an acceleration factor of 2 was used.

Before fMRI preprocessing, volumes were identified as outliers on signal intensity using Mahalanobis distances (3 s.d.), and dummy regressors were included as nuisance covariates in the first-level models. Functional images were corrected for differences in the acquisition timing of each slice and were motion-corrected (realigned) using SPM8. Twenty-four head motion covariates per run were entered into each first-level model (displacement in six dimensions, displacement squared, derivatives of displacement and derivatives squared). Structural T1-weighted images were then coregistered to the mean functional images using SPM8’s iterative mutual information-based algorithm. Coregistered, high-resolution structural images were warped to Montreal Neurologic Institute (MNI) space (avg152T1.nii); these warping parameters were applied to the functional data, normalizing it to MNI space, and interpolated to 2 × 2 × 2 mm^3^ voxels. Finally, functional images were smoothed with an 8 mm FWHM Gaussian kernel. A 220 s high-pass filter was applied during the first-level analysis.

#### fMRI analyses.

Our analyses focused on the period of listening to biography reminders, which were briefer (11.5 s) than the initial biography listening period (33.5 s) and more proximal to charitable donation decisions. We estimated a general linear model using SPM8 for each participant, including the nuisance covariates generated in preprocessing and two regressors of interest: listening to biography reminders (11.5 s) and the charitable donation decision period (5 s), each convolved with the standard hemodynamic response function. The jittered-duration inter-trial interval served as the model intercept. We then computed contrast images for the (listen-baseline) comparison for every subject at pre- and post-intervention. We subtracted the pre-intervention image from the post-intervention image to estimate pre-to-post-intervention changes in brain responses to stories of suffering.

We tested two comparisons—CM *vs* Familiarity and CM *vs* OxyPla—to identify the specific effects of CM on brain activity. For archival purposes, we also estimated the OxyPla *vs* Familiarity comparison to characterize placebo effects on brain activity; these results are reported in the supplementary material (Supplementary Table S1, Figure S6).

#### Region of interest selection.

Previous CM studies have reported effects primarily in two sets of brain regions: (i) elements of networks involved in regulation of motivated behavior, including the OFC, VTA and NAc (Klimecki *et al.*, 2014; Singer and Klimecki, 2014; Engen and Singer, 2015b), which are also part of the mesolimbic dopaminergic pathway, and (ii) regions linked to construction of conceptual models and mentalizing, including the dmPFC and the TPJ (Lutz *et al.*, 2008; Mascaro *et al.*, 2013; Weng *et al.*, 2013; Ashar *et al.*, 2016a; Valk *et al.*, 2017). VTA and NAc definitions were taken from a high-resolution subcortical atlas (Pauli *et al.*, 2018). A mask for the OFC was adopted from a recent multimodal cortical parcellation (region ‘OFC’ in Glasser *et al.*, 2016). Masks for the dmPFC and TPJ were created from 10 mm spheres around peak coordinates reported in prior CM studies, with symmetrical (mirrored) spheres in both hemispheres (Mascaro *et al.*, 2013 for dmPFC [−9, 50, 37]; Weng *et al.* (2013) for TPJ [46, −62, 36]).

We tested for group differences in two masks, one covering each set of regions, at a threshold of *P* < 0.05 familywise error rate (FWER) corrected. Correction was performed using permutation testing (10 000 permutations) in FSL randomize v2.9 using threshold-free cluster-enhancement (Smith and Nichols, 2009). Given *a priori* directional hypotheses from prior literature, we tested for CM *vs* control increases (not decreases) in brain responses to suffering others. In clusters showing a significant group difference, we conducted a robust regression within CM participants testing whether change in brain activity correlated with (i) changes in FAS scores and (ii) changes in charitable donations.

#### Whole-brain gray matter analyses.

To more broadly characterize intervention effects on brain function, we conducted a whole-brain robust regression (Wager *et al.*, 2005) estimating group differences in pre-to-post-intervention changes in brain activity. We also conducted a robust regression estimating average pre-to-post-intervention changes within each group, to characterize absolute pre-to-post-intervention changes independently for each group. Analyses were conducted two-tailed within a gray matter mask, as results in white matter and cerebrospinal fluid are unlikely to reflect changes in neuronal activity. As part of a standard quality-control process, we also verified that there were no unexpected regions of activation in white matter and ventricle spaces that would be consistent with artifacts. We applied an exploratory threshold of *P *< 0.001 uncorrected and 10 or more contiguous voxels. This is a commonly used threshold providing a balance between type I and type II error rates. It is most appropriate for hypothesis-generation purposes and aggregation of findings across studies in meta-analyses, especially in whole-brain analyses with limited sample sizes and moderately sized effects (Lieberman and Cunningham, 2009; Woo *et al.*, 2014).

Neuroanatomical labeling was conducted using the freely available CanlabCore tools, which pool anatomical labels from across number of published atlases (see https://canlabcore.readthedocs.io/en/latest/moduleslist.html#@region.table) and with reference to the a recent histological atlas (Amunts *et al.*, 2020). Contrast images for each subject at each time point, behavioral outcomes and code for all analyses are publicly available at https://github.com/yonestar/effects_of_CM_on_brain.

#### Intervention effects on brain models of empathic care and distress.

We tested the effect of the interventions on two previously published whole-brain patterns associated with two distinct emotional responses to suffering others: empathic care—an affiliative, tender emotional response—and empathic distress, a high-arousal negatively valenced emotional response (Ashar *et al.*, 2017a). We hypothesized that the CM group would exhibit pre-to-post-intervention increases in the empathic care brain model relative to control conditions. A secondary hypothesis was of increases in the empathic distress brain model for CM *vs* Familiarity, driven primarily by decreased distress for Familiarity participants, potentially paralleling the previously published behavioral outcomes.

We computed the cosine similarity between the care and distress models and each subjects’ contrast images at both pre- and post-intervention. Cosine similarity is a normalized measure of similarity between two vectors (i.e. brain patterns). It is equivalent to both (i) the dot product after normalizing whole-brain image intensity and (ii) Pearson’s correlation without mean-centering images and can be used for assessing multivariate pattern expression in test images (e.g. Mitchell *et al.*, 2008; Kragel *et al.*, 2018). We computed a pre-to-post-intervention change score for each participant for both the empathic care and empathic distress models and submitted these to two-sample *T*-tests of CM *vs* OxyPla and CM *vs* Familiarity group differences.

#### Tests of CM protecting against decreased brain responses to suffering others.

**Fig. 1.**
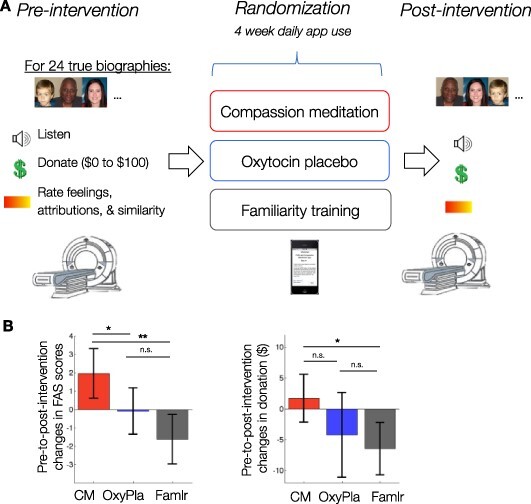
Study design and behavioral results. (A) Participants completed a compassion task during functional MRI, were randomized to a compassion meditation intervention or a control intervention and then returned for a second functional MRI session. (B) Pre-to-post-intervention changes in charitable donations and in a composite index of compassion-related feelings and attributions (‘FAS scores’, see main text), as previously reported (Ashar *et al.*, 2016b). Error bars show 95% CI; * *P *< 0.05, ** *P *< 0.01, *** *P *< 0.001.

In the behavioral outcomes, we observed significant, unexpected decreases in the Familiarity group for compassion-related feelings and attributions (measured with FAS scores) and for charitable donations (Figure 1B). CM protected against these decreases (i.e. caused relative increases compared with Familiarity), suggesting that it may buffer against decreases in compassion during repeated exposures to suffering. We tested for a neural parallel of these behavioral effects.

In 5-fold cross-validated analyses, we selected the 5% of voxels exhibiting the largest pre-to-post-intervention decreases within a training subset of Familiarity participants. We then extracted the mean pre-to-post-intervention change in those voxels from the held-out participants, and we computed the standardized mean difference (Hedge’s *g*) between Familiarity and CM participants in pre-to-post-intervention change scores. This provided a minimally biased procedure for identifying the largest neural decreases in the Familiarity group and testing for a buffering effect of CM in independent, held-out participants. Results were compared with a null distribution created by permuting the group labels and repeating the analysis 10 000 times.

## Results

### Behavioral results

We first summarize the previously published (Ashar *et al.*, 2016b) behavioral outcomes from the trial, to provide a context for the fMRI outcomes that are the focus of this manuscript.

#### Participant compliance, attention to task and expectations.

Intervention compliance, as logged by the intervention iPod applications, was high across groups. At the same time, CM participants completed their daily tasks significantly less frequently than other participants (out of 28 possible days, CM: *M* = 20.76 days, 95% CI [18.81, 22.71]; OxyPla: *M* = 26.78 days, 95% CI [25.38, 28.18]; Familiarity: *M* = 25.39 days, 95% CI [24.10, 26.68]; *F*(2, 53) = 17.15, *P* < 0.001). Similarly, performance on the daily attention-to-task questions was near ceiling across groups, *M *= 98% correct, although there were significant group differences in correct responding, *F*(2, 54) = 6.00, *P *= 0.004, which were driven by slightly lower performance in the CM group, *M*_CM_* = *95% correct, 95% CI = [0.93, 0.98].

Pre-intervention expectations of increased compassion were moderately high across groups, *M *= 5.00 on a 0 to 10 scale, 95% CI [4.40, 5.60]. There were significant group differences between the three groups, *F*(2, 49) = 6.06, *P *= 0.004. This was driven by lower expectations in the Familiarity condition, *M* = 3.69 out of 10, 95% CI [2.49, 4.88]. Expectations in the Familiarity condition were statistically lower relative to both the CM condition, *T*(32) = 3.28, *P* = 0.003, 95% CI [0.86, 3.66], and the OxyPla condition, *T*(31) = 1.97, *P *= 0.06, 95% CI [−0.05, 2.91]. Direct comparison of expectations in the CM and OxyPla conditions showed no statistical difference, *T*(33) = 1.32, *P *= 0.19. At the study end, all but one of the OxyPla participants reported believing that they had been taking true oxytocin.

#### Changes in compassion and donation.

Relative to Familiarity participants, CM participants increased in both FAS scores and charitable donations from pre- to post-intervention (Hedge’s *g*_FAS_ = 1.17, 95% CI [0.69, 1.76], and *g*_donation_ = 0.89, 95% CI [0.35, 1.47], *M*_donation difference_ = $8.17, 95% CI [$2.22, $14.11]). Relative to OxyPla participants, CM participants significantly increased in FAS scores, *g*_FAS_ = 0.69, 95% CI [0.10, 1.34], but the difference in donations was not statistically significant, *g*_donation_ = 0.48, 95% CI [−0.14, 0.97], *M*_donation difference_ = $5.95, 95% CI [$-2.33, $14.23]. Examining patterns of absolute pre-to-post-intervention change within group, we found that CM participants increased in FAS scores, *g* = 0.61, 95% CI [0.27, 1.03], and did not statistically change in donation amounts, *g* = 0.19, 95% CI [−0.27, 0.60]. OxyPla participants did not statistically change in either outcome, *g*_FAS_ = −0.03, 95% CI [−0.56, 0.41], *g*_donation_ = −0.27, 95% CI [−0.58, 0.22]. Familiarity participants decreased in both outcomes, *g*_FAS_ = −0.53, 95% CI [−0.95, −0.18], *g*_donation_ = −0.67, 95% CI [−1.06, −0.36] (Figure 1B). Overall, this profile of behavioral results suggests that (i) Familiarity participants decreased in compassion over time, (ii) CM buffered against this decrease and led to increased compassion and (iii) the effects of CM were partly but not fully attributable to placebo.

### fMRI results

#### Region of Interest (ROI) analyses.

**Fig. 2.**
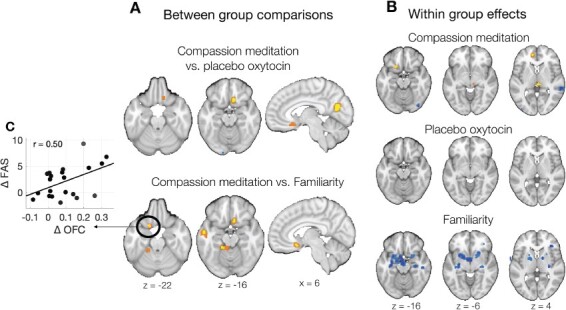
Effects of CM *vs* controls on brain responses to audio-video narratives describing suffering others. (A) Group differences in pre-to-post-intervention changes. (B) Absolute pre-to-post-intervention changes within each intervention condition. Circled clusters are significant at a threshold of *P* < 0.05 FWER-corrected. Non-circled clusters meet an exploratory threshold of *P *< 0.001 uncorrected with 10 contiguous voxels. Adjacent voxels meeting a threshold of *P *< 0.005 uncorrected shown in orange/light blue for visualization purposes. Yellow/orange areas show increases and blue areas show decreases. (C) Among CM participants, increases in mOFC activity positively correlated with increased FAS scores, our primary measure of compassion-related emotions and appraisals (e.g. reduced blame and increased tenderness; see text for details).

For the CM *vs* Familiarity comparison, we observed significantly increased brain responses to suffering others in the mOFC, *P *= 0.03 FWER-corrected (Figure 2A, region center *x y z* = [−18 12 –22] mm in the MNI space). Individual differences in pre-to-post-intervention increases in mOFC activity were positively correlated with increases in FAS scores, *r*(19) = 0.50, *P *= 0.04 (Figure 2C), and not with charitable donations, *r*(19) = 0.28, *P *> 0.2. No CM *vs* Familiarity group differences were observed in the dmPFC or TPJ. No CM *vs* OxyPla group differences were observed, FWER-corrected within the regions of interest.

#### Whole-brain gray matter analyses.

In whole-brain analyses applying an exploratory threshold, the CM *vs* Familiarity comparison yielded three clusters with relatively increased activity for the CM condition: the mOFC, left superior temporal sulcus and left parahippocampal cortex. The CM *vs* OxyPla comparison yielded three clusters with relatively increased activity in the CM condition: the mOFC and two occipital areas (Figure 2, coordinates listed in Supplementary Table S1).

For within-group pre-to-post-intervention changes, CM participants exhibited increased brain responses to suffering others in the mOFC, mPFC, midbrain and the right cerebellum. OxyPla participants exhibited no significant increases in brain activity over time and some decreases in V3 and lateral prefrontal areas. Familiarity participants exhibited decreases across several prefrontal and subcortical structures, including mOFC, insula, superior temporal sulcus, amygdala, hippocampus, hypothalamus, putamen and globus pallidus (Figure 2B, coordinates listed in Supplementary Table S2).

Three-dimensional interactive images of all results are available at https://neurovault.org/collections/4766/, and figures showing full-brain results are provided in Supplementary Figures S1–S6.

#### *Effects on.* a priori *brain models of empathic care and distress.*

Effects of the intervention were in the expected direction, with CM participants exhibiting increases in the neural signature response (i.e. cosine similarity or pattern expression) for both empathic care and empathic distress relative to both OxyPla and Familiarity participants. However, group differences were not statistically significant, *P*s ranging from 0.12 to 0.72.

#### Tests of CM protecting against decreased brain responses to suffering others.

We found a small ‘buffering’ effect of CM *vs* Familiarity in the expected direction, *g *= 0.22, which did not achieve statistical significance, *P *> 0.4. Although pre-to-post-intervention decreases across a range of subcortical regions were observed in Familiarity participants (Figure 2B), it is unclear to what extent CM protected against these.

#### Post-hoc power analyses.

We observed an effect of magnitude *d *= 0.6 in the mOFC for the CM *vs* OxyPla comparison. *Post-hoc* analyses revealed that a sample size of *N *= 45 per group is needed for 80% power for an effect of this size, with two-tailed α = 0.05, indicating that future studies with resources for larger samples are needed.

## Discussion

CM is a promising intervention for enhancing compassionate responding to suffering others. Here, we investigated the neurobiological mechanisms supporting the effects of CM. We compared a 4-week smartphone application–based CM intervention with two control conditions—placebo oxytocin (OxyPla) and a Familiarity control—to more closely isolate the specific effects of CM on brain function. We found significant CM *vs* Familiarity increases in mOFC responses to audiovisual narratives depicting suffering others, with CM *vs* OxyPla effects on mOFC function also observed at an exploratory threshold. Among CM participants, individual differences in mOFC increases positively correlated with increases in compassion-related feelings and attributions (e.g. increased tenderness and reduced blame). We note that the mOFC region is adjacent to and potentially functionally overlaps with other anatomical labels, including subcallosal anterior cingulate. This area is also often included in the ventromedial prefrontal cortex (vmPFC), a broad zone encompassing multiple cortical regions (Koban *et al.*, 2021).

Much research across humans and other species has strongly linked the mOFC—and larger, overlapping vmPFC zone—to a variety of forms of motivated behavior (Haber and Knutson, 2010), particularly when motivated behavior requires the construction of cognitive frames or schemas that supply personal meaning to events and actions (Roy *et al.*, 2012; Barrett, 2017; Ashar *et al.*, 2017b). In the interpersonal context, mOFC activity has been linked to empathic care for suffering others (Ashar *et al.*, 2017a), altruistic giving (Cutler and Campbell-meiklejohn, 2019) and the perceived value of others (Watson and Platt, 2012; Zerubavel *et al.*, 2015; Parkinson *et al.*, 2017; Morelli *et al.*, 2018). Damage to the vmPFC and OFC is associated with increased utilitarian moral judgments (Koenigs *et al.*, 2007), and pathological lack of empathy, guilt and remorse is associated with reduced vmPFC and OFC responses to stimuli depicting suffering others (Decety *et al*., 2013). Medial prefrontal representations also shift based on active perspective-taking processes, such that others’ preferences are represented in more ventral mPFC areas when choosing on their behalf (Nicolle *et al.*, 2012). Taken together, the engagement of the mOFC by CM may reflect an increased value placed on the welfare of suffering others, increased affiliation and/or increased empathic care. Integrative theories of vmPFC–OFC function suggest that this is a conceptual process integrating information about another person with a representation of one’s well-being.

Our findings are in broad agreement with a number of previous CM findings. Engen and Singer (2015b) found that practicing CM during viewing of emotional videos activates the mOFC, as compared with practicing cognitive reappraisal strategies. Randomized trials that compared CM with empathy and memory training have found that CM increases activity in mOFC areas overlapping or adjacent to the mOFC cluster reported here (Klimecki *et al.*, 2012, 2014). Further, long-term meditators more strongly engage the mOFC when practicing CM relative to novice practitioners (Engen *et al.*, 2018). Our findings are encouraging for the generalizability of the mOFC as an important neurobiological mechanism of CM. Relative to prior studies, ours tested a different CM implementation (including different specific CM practices, different delivery format and different intervention length) in a different participant population (participants from Germany *vs* the USA). Since we did not ask CM participants to engage in CM practices during the scan, our findings also suggest that CM effects likely transfer to non-meditative states.

A recent debate has focused on whether CM primarily engages more cognitive or affective processes (Dahl *et al.*, 2015, 2016; Engen and Singer, 2015a). Yet, cognitive and affective processes interact to promote empathy and compassion—both are important (De Waal, 2008; Zaki and Ochsner, 2012; Kanske *et al.*, 2016). While mOFC function has been typically more closely related to affective than cognitive empathy processes, both these processes are likely intertwined in the mOFC (Roy *et al.*, 2012; Barrett, 2017; Ashar *et al.*, 2017b). Relatedly, our results cannot disambiguate between processes that are activated during the practice of CM *vs* the outcome of CM (Dahl *et al.*, 2016). CM also refers to a family of meditation practices, and there may be substantial variability in mechanisms among different CM implementations, with some engaging more cognitive processes. For example, cognitively based compassion training uses a ‘cognitive, analytic approach to challenge one’s unexamined thoughts and emotions toward other people… various arguments are examined that challenge one’s common sense notion of other people as falling into the categories of “friend, enemy and stranger”’ (Pace *et al.*, 2009).

What are the specific effects of CM on brain function, above and beyond placebo and familiarity effects? We found a significant effect of CM *vs* Familiarity in mOFC responses to suffering others, with weaker CM *vs* OxyPla effects observed at an exploratory threshold. These results parallel the self-reported and behavioral outcomes, in which we observed significant group differences for the CM *vs* Familiarity comparison, which were attenuated for the CM *vs* OxyPla comparison. Overall, these findings indicate effects of CM on brain function are not explained by familiarity effects and are partly explained by placebo effects. Another investigation comparing mindfulness meditation to sham (placebo) meditation similarly reported specific effects of mindfulness on brain function, in this case, in responses to painful experimental stimuli (Zeidan *et al.*, 2010, 2015).

A critical meditation research challenge is developing well-matched control conditions (MacCoon *et al.*, 2012; Davidson and Kaszniak, 2015). Our placebo manipulation succeeded in generating pre-intervention expectations of increased compassion similar to those reported in the CM group, and all but one participant reported believing they had been taking verum oxytocin. At the same time, the placebo oxytocin nasal spray likely engaged an overlapping but distinct set of expectations and associations relative to CM. For example, oxytocin is often portrayed as the ‘love hormone’ in popular culture—a reference related to but different from compassion—and participants might have expected different time courses of intervention effects from nasal spray *vs* meditation. Designing controls with tightly matched expectations is a major challenge for psychological interventions (Boot *et al.*, 2013).

Additionally, the two CM comparison conditions included here were not matched on other non-specific factors, such as time spent engaged in the intervention each day. CM participants completed the CM intervention less often than the control interventions (although CM compliance was still relatively high overall—encouraging for a smartphone CM implementation). This relatively less-frequent engagement with the CM app may have either detracted from its efficacy (due to a lower ‘dose’ of CM) or enhanced it (if more exposure to suffering reduces compassion). Future studies including more closely matched control groups will continue to refine our understanding of the ‘active ingredients’ of CM.

We cannot generalize our findings to participants who are unwilling to donate to charity, as these subjects were excluded. It is unknown whether these people differ in their response to CM interventions. Our results also have limited generalizability to groups underrepresented in our sample, including racial/ethnic minorities. Racial/ethnic minority groups have been broadly underrepresented in meditation research (Waldron *et al.*, 2018), marking an important area for future research.

We observed decreased activity in Familiarity participants across a range of prefrontal and subcortical regions, including the mOFC, amygdala, hippocampus, insula and more. These decreases were not observed in the CM and/or placebo groups, suggesting a potential ‘buffering effect’ of these interventions against decreased compassion, as seen in the behavioral data. However, we did not detect a significant group by time interaction in these regions (besides in the mOFC), and direct tests of this buffering hypothesis failed to reach statistical significance. Alternately, reduced responsivity to others’ suffering might be a positive outcome in some ways and in some contexts, since heightened emotional responses to suffering can lead to professional ‘burnout’ (Maslach *et al.*, 2001; Shanafelt *et al.*, 2017). In support of this, mindfulness-based interventions have been found to reduce brain responses to emotionally distressing stimuli, which was interpreted as a positive outcome (Desbordes *et al.*, 2012). Future studies are needed to better understand how reduced brain responses to suffering others may alternately reflect equanimity or diminished compassion.

Our exploratory analyses additionally revealed several additional regions of potential interest. As these regions did not survive correction for multiple comparisons, further studies might conduct more targeted investigations of their role in CM. One finding of interest is the increased response in visual cortex for CM *vs* OxyPla, which might reflect increased visual engagement with the stimuli depicting suffering others. A second finding of interest is the increased parahippocampal response for CM *vs* Familiarity, which might reflect increased recruitment of memory- or imagination-related processes during engagement with suffering others, consistent with recent work on the importance of the episodic memory system for compassion (Gaesser *et al.*, 2019a,b).

In sum, our findings suggest that compassion training can increase psychological, behavioral and brain processes that support empathic care and prosocial behavior. Compassion training rests of the assumption that compassion is a skill and a choice (Halifax, 2012; Zaki, 2014). Studies have found that it is often a difficult choice—engaging with others’ suffering is effortful and often avoided (Cameron, 2017; Cameron *et al.*, 2019). Yet, compassion training programs can teach skills for engaging with others’ suffering without becoming overwhelmed, cultivating a sensitive and sustainable approach for enacting compassion in daily lives. Investigating the psychological and neurobiological mechanisms of compassion training interventions will help us better understand these processes, promoting their development and dissemination.

## Supplementary Material

nsab052_Supp
