# Erratum to: effects of compassion training on brain responses to suffering others

**DOI:** 10.1093/scan/nsab068

**Published:** 2021-06-12

**Authors:** Yoni K Ashar, Jessica R Andrews-Hanna, Joan Halifax, Sona Dimidjian, Tor D Wager

**Affiliations:** Department of Psychology and Neuroscience, University of Colorado Boulder, Boulder, CO 80309, USA; Department of Psychiatry, Weill Cornell Medical College, New York, NY 10075, USA; Department of Psychology, University of Arizona, Tucson, AZ 85721, USA; Upaya Institute and Zen Center, Santa Fe, NM 87501, USA; Department of Psychology and Neuroscience, University of Colorado Boulder, Boulder, CO 80309, USA; Renee Crown Wellness Institute, University of Colorado Boulder, Boulder, CO 80309, USA; Department of Psychology and Brain Sciences, Dartmouth College, Hanover, NH 03755, USA

In the originally published version of this manuscript, there was an error in the title.

The incorrect title was *Effects of compassion training on brain responses to suffering other*.

The correct title is *Effects of compassion training on brain responses to suffering others*.

This has now been corrected.

